# Efficacy and Safety of Transdermal Buprenorphine versus Oral Tramadol/Acetaminophen in Patients with Persistent Postoperative Pain after Spinal Surgery

**DOI:** 10.1155/2017/2071494

**Published:** 2017-09-13

**Authors:** Jae Hyup Lee, Jin-Hyok Kim, Jin-Hwan Kim, Hak-Sun Kim, Woo-Kie Min, Ye-Soo Park, Kyu-Yeol Lee, Jung-Hee Lee

**Affiliations:** ^1^Department of Orthopedic Surgery, Seoul National University College of Medicine, SMG-SNU Boramae Medical Center, Seoul, Republic of Korea; ^2^Department of Orthopedic Surgery, Sanggye Paik Hospital, Seoul, Republic of Korea; ^3^Department of Orthopedic Surgery, Ilsan Paik Hospital, Gyeonggi-do, Republic of Korea; ^4^Department of Orthopedic Surgery, Gangnam Severance Hospital, Yonsei University, Seoul, Republic of Korea; ^5^Department of Orthopedic Surgery, Kyungpook National University Hospital, Daegu, Republic of Korea; ^6^Department of Orthopedic Surgery, Guri Hospital, Hanyang University College of Medicine, Gyeonggi-do, Republic of Korea; ^7^Department of Orthopedic Surgery, Dong-A University Hospital, Busan, Republic of Korea; ^8^Department of Orthopedic Surgery, Graduate School, College of Medicine, Kyung Hee University, Seoul, Republic of Korea

## Abstract

**Purpose:**

Control of persistent pain following spinal surgery is an unmet clinical need. This study compared the efficacy and safety of buprenorphine transdermal system (BTDS) to oral tramadol/acetaminophen (TA) in Korean patients with persistent, moderate pain following spinal surgery.

**Methods:**

Open-label, interventional, randomized multicenter study. Adults with persistent postoperative pain (Numeric Rating Scale [NRS] ≥ 4 at 14–90 days postsurgery) were enrolled. Patients received once-weekly BTDS (*n* = 47; 5 *μ*g/h titrated to 20 *μ*g/h) or twice-daily TA (*n* = 40; tramadol 37.5 mg/acetaminophen 325 mg, one tablet titrated to 4 tablets) for 6 weeks. The study compared pain reduction with BTDS versus TA at week 6. Quality of life (QoL), treatment satisfaction, medication compliance, and adverse events (AEs) were assessed.

**Findings:**

At week 6, both groups reported significant pain reduction (mean NRS change: BTDS −2.02; TA −2.76, both *P* < 0.0001) and improved QoL (mean EQ-5D index change: BTDS 0.10; TA 0.19, both *P* < 0.05). The BTDS group achieved better medication compliance (97.8% versus 91.0%). Incidence of AEs (26.1% versus 20.0%) and adverse drug reactions (20.3% versus 16.9%) were comparable between groups.

**Implications:**

For patients with persistent pain following spinal surgery, BTDS is an alternative to TA for reducing pain and supports medication compliance. This trial is registered with Clinicaltrials.gov: NCT01983111.

## 1. Introduction 

Spinal stenosis is a common cause of pain affecting the back and legs due to narrowing of the spinal canal or neural foramen. Decompression surgery is often performed to treat this condition by removing excess bone, thickened ligaments, degenerate disc material, and other fibrous tissue causing the stenosis. Sometimes, a fusion procedure is performed to fuse two adjoining vertebrae together to make the spine more stable in the case of severe spinal stenosis or unstable spondylolisthesis [1]. The incidence of spinal stenosis and other degenerative spine conditions increases with age, and spinal fusion surgery is often required to treat such conditions. Patients who have undergone lumbar spinal surgery are vulnerable to adverse consequences from unrelieved or undertreated postoperative pain after surgery. This is because pain assessment is often fraught with problems arising from difficulties in measuring and reporting pain intensity, and, in older patients, the presence of cognitive impairment [2]. Inadequate postoperative pain management increases the risk of complications from the spinal surgery and reduces the mobility of patients and may even delay the rehabilitation and recovery process due to the pain [3]. Major spinal surgery is associated with moderate-to-severe pain on the first day after surgery (median score of 7 on the Numeric Rating Scale [NRS], interquartile range: 5–8) [4]. With analgesic treatment, pain intensity generally declines from moderate or severe to mild levels over the first 24–48 h after surgery, but a proportion of patients may continue to experience moderate or severe pain beyond this period [5–7]. During the immediate postoperative period, strong opioid analgesics may be used for relief of acute pain. Strong opioids are highly effective and quick-acting in managing pain. However, they have various adverse effects and therefore their benefits should be weighed against this risk, especially for long-term use [8]. This limits their prolonged use for managing subacute pain (1–3 months after surgery) [9] and prompts research effort to find better and more suitable analgesics for control of persistent postoperative pain. In particular, the transition from acute to subacute and chronic pain following surgery is not well studied, and it has been pointed out that further attention and research are warranted in this area [10, 11].

Although considered a weak opioid because of its much lower affinity for the *μ*-receptor, tramadol may be prescribed for patients with complicated pain etiology owing to its dual mechanism of action: binding to *μ*-receptors as well as inhibiting serotonin and norepinephrine reuptake in the central nervous system [12]. Tramadol has a broad range of therapeutic indications, from acute to chronic pain, and has been used as a first-line treatment in musculoskeletal system disorders, for relief of persistent postoperative pain, as well as various types of chronic pain [13]. However, oral formulations of tramadol have a number of systemic side-effects such as headache, sleep disturbance, constipation, vomiting, hydrodipsomania, sweating, nausea, and dizziness [14] and, like other opioids, prolonged use may be associated with dependence and abuse [15]. To manage persistent postoperative pain, opioid analgesics such as tramadol have been used together with non-opioid analgesics such as acetaminophen to enhance their analgesic effects and improve quality of life (QoL) for patients [13, 16, 17].

Buprenorphine is a synthetic opioid analgesic used in the management of postoperative pain. A review of the buprenorphine transdermal patch found that it was as effective as other opioids such as oral morphine, oxycodone, and fentanyl in relieving pain and that buprenorphine could be used to achieve the same effect at lower dose equivalents [18]. Studies in patients with chronic postoperative pain suggest that CNS sensitization is also reduced or absent with buprenorphine compared with other agents [19]. Buprenorphine can be administered via various routes. A once-weekly patch for transdermal application is available as a dosing regimen that can maintain an analgesic effect equivalent to tramadol [20]. While the buprenorphine transdermal system (BTDS) has been investigated in a number of studies and, in particular, for the management of chronic pain [18, 21–23], its efficacy and safety for the relief of persistent postoperative subacute pain have not been extensively studied. Owing to the slower-acting pharmacological profile of buprenorphine, BTDS cannot be used for immediate pain relief or as a substitute for short-acting analgesics [19], but may have potential for use in managing persistent postoperative pain. For patients with multiple comorbidities who face an increasing number of orally administered drugs, the use of once-weekly BTDS offers the potential benefits of reduced pill burden and greater convenience.

In a proportion of patients, postoperative pain can be serious and may persist for prolonged periods (several weeks up to several months) [24]. Achieving adequate postoperative pain control remains an unmet need during the subacute phase, where pain is present for at least 6 weeks but less than 3 months [25]. Research on the use of analgesics for subacute pain is limited, and a literature search revealed no studies comparing transdermal buprenorphine to oral tramadol/acetaminophen for the treatment of subacute postoperative pain. This study reports on a comparison of transdermal buprenorphine (BTDS) treatment with oral tramadol/acetaminophen (TA) for reducing pain in patients with persistent postoperative pain following lumbar spinal surgery.

## 2. Materials and Methods

This was a multicenter, open-label, interventional, randomized, noninferiority study conducted at 8 medical institutions in South Korea. It was conducted in accordance with Korea Good Clinical Practice (KGCP) [26] and International Conference on Harmonisation (ICH) [27] guidelines, complied with the rights and safety of subjects under the Declaration of Helsinki, and was approved by the Institutional Review Boards (IRB) of all eight institutions. All patients provided written informed consent prior to participating in the study.

### 2.1. Study Design

**Figure 1:**
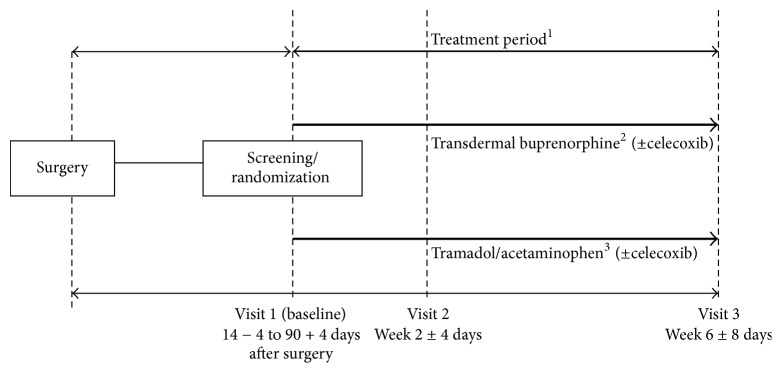
*Study design and visit schedule*. ^1^Unscheduled visits could be conducted at any time during the treatment period. Domperidone maleate was concurrently prescribed for 2 weeks from initiation of the study drug administration. Subsequently, continued use was determined at the discretion of the investigator. ^2^Permitted study drug dose escalation: transdermal buprenorphine (BTDS) 5 *μ*g/h to 10 *μ*g/h to 15 *μ*g/h or 20 *μ*g/h. ^3^Permitted comparator drug dose escalation: tramadol/acetaminophen (TA; tramadol 37.5 mg/acetaminophen 325 mg) 1 tablet × 2 doses per day to 2 tablets × 2 doses per day to 3 tablets × 2 doses per day or 4 tablets × 2 doses per day. BTDS: buprenorphine transdermal system (NORSPAN); TA: tramadol/acetaminophen.

The study had two arms: the BTDS group which received the study drug, buprenorphine transdermal patch (NORSPAN®), and the TA group which received the comparator drug, tramadol 37.5 mg/acetaminophen 325 mg tablet (Ultracet® ER Semi Tab), which is commonly used in Korea (Figure 1). There were 3 visits: Visit 1 (baseline, at week 0: 14–90 ± 4 days after surgery), Visit 2 (week 2 ± 4 days), and Visit 3 (week 6 ± 8 days), as shown in Figure 1. At Visit 1, patients were randomized (1 : 1) to receive either BTDS or TA, based on computer-generated randomization lists. The initial dose (baseline) for BTDS was 5 *μ*g/h for 7 days and this was increased to 10, 15, or 20 *μ*g/h during the study, depending on the subject's pain intensity and at the discretion of the investigator. The initial dose for the TA group was 1 tablet twice daily and was increased to 2, 3, or 4 tablets twice daily depending on the subject's pain intensity and at the discretion of the investigator. The rescue medication, when needed, was celecoxib (Celebrex® 200 mg capsules).

### 2.2. Patients

The study enrolled patients aged ≥20 years who had lumbar fusion surgery (1-2 level fusion) and experienced persistent pain (numerical rating scale, NRS [28] ≥ 4, 0–10 points, with 0 points = no pain, mild pain = 1–3, moderate pain = 4–6, and severe pain = 7–10) 14–90 days after surgery. Key exclusion criteria included use of strong opioids, buprenorphine or tramadol/acetaminophen < 1 week prior to trial participation; current use of CNS depressants or muscle relaxants; postoperative complications; symptoms of acute pain after lumbar fusion or a pain relief profile characterized by rapidly changing analgesic requirements; major pain not attributable to spinal disease; ongoing anticancer treatment that could affect pain assessment; clinically significant respiratory, cardiovascular, renal, or hepatic impairment; any hypersensitivity, allergy, or other contraindication to the use of buprenorphine, tramadol/acetaminophen or celecoxib, or domperidone; administration of other investigational products at enrollment or within 30 days of trial participation; history of drug abuse or medical history of narcotic or drug dependence. Pregnant women or those of childbearing potential who were unwilling or unable to use appropriate contraception during the study were also excluded.

Informed patient consent was obtained prior to the start of study. All demographic information, medical history, physical examination, and vital signs were recorded. Body temperature, pulse, and blood pressure were measured at every visit and clinical laboratory tests were conducted at Visits 1 and 3. Pregnancy tests were conducted for women of childbearing potential at Visits 1 and 3 with a human chorionic gonadotropin (hCG) urine test.

### 2.3. Study Assessments

#### 2.3.1. Pain Intensity

Subjects were asked to indicate the intensity of pain symptoms using NRS over the last 24 hours at every visit. At Visit 1 (baseline), pain assessment was conducted prior to investigational product administration. The primary efficacy evaluation was based on the change in the pain intensity from baseline to Visit 3 (week 6). Noninferiority of BTDS to TA for pain reduction at week 6 was evaluated.

#### 2.3.2. Quality of Life and Treatment Satisfaction

At baseline and week 6, subjects were also asked to assess their health-related quality of life (QoL) using the EuroQol Group 5-Dimension Self-Report Questionnaire (EQ-5D-3L [29], Korean version), consisting of 5 items, that is, mobility, self-care, usual activities, pain/discomfort, and anxiety/depression. Each item was evaluated at 3 levels. The total EQ-5D index score was computed using a score table (Table 1) as follows: 1 − 0.081 − (score according to item and level) − 0.269 (if there is at least one Level 3 item).

At Week 2 and Week 6, investigators evaluated the overall satisfaction with treatment using a 7-point Clinical Global Impression of Change scale (CGIC, 1 = very much improved to 7 = very much worse) [30, 31]. At the same time-points, subjects were also asked to evaluate their overall satisfaction with the treatment since the last visit using a 7-point Patient Global Impression of Change [32] scale (PGIC, 1 = very much improved to 7 = very much worse). The changes from baseline to week 6 in the EQ-5D, CGIC, and PGIC scores were calculated.

#### 2.3.3. Medication Use and Compliance

Subjects were instructed to return all unused medication at every visit. Actual dosing was determined by comparing the amount of unused study or comparator drug returned by the subject against the prescribed dose. The final dose of the investigational product at week 6 was also compared to the initial dose at baseline. The compliance rate was computed as the “total number of doses actually administered/total number of doses prescribed.” A compliance rate of ≥80% was deemed acceptable. The total administered dose of rescue medication (celecoxib) for each group was computed as total number of doses of rescue medication administered × 200 mg.

#### 2.3.4. Safety

Subjects were asked to report all adverse events (AEs). This information was also obtained from the subject via interview during scheduled visits in the study period. The AE information included the onset and resolution dates, AE severity, action taken and outcome, and causal relationship with the investigational product or suspected drugs other than the investigational product.

### 2.4. Statistical Analysis

Noninferiority of BTDS to TA in terms of pain intensity reduction was evaluated by comparing the lower limit of the one-sided 97.5% confidence interval (CI) for the between-group difference in the change in pain scores from baseline to week 6 with a prespecified margin of 1.5, as described by Karlsson and Berggren [20]. In that study, which compared BTDS with tramadol in patients with chronic osteoarthritis pain, the mean pain score (on the BS-11 scale [33]; range 0–10) for tramadol was 6.39 ± 1.58 at baseline; and after 12 weeks of treatment, the mean score decreased to 3.80 ± 2.20, giving a change of −2.59 ± 2.20 units [20]. Sample size estimation was performed accordingly: in order to detect differences with ≥90% power at a level of significance of 0.025 (one-sided), the sample size was computed as 47 subjects per group. Allowing for a dropout rate of 30%, it was estimated that 68 subjects would need to be randomized to each of the 2 treatments (total of 136 subjects). Notwithstanding some differences in study duration and patient population, estimates from [20] were used in designing the present study, as it appeared the most similar in terms of treatments and pain context (BTDS versus tramadol in nonacute pain).

The full analysis set (FAS), that is, patients who received at least one dose of study or comparator treatment, was used for the efficacy and QoL analyses and supported by additional analyses on the per-protocol (PP) set, that is, patients who completed the study without major protocol deviations. The FAS was also used for medication use and compliance analyses. Safety analysis was performed on the enrolled patients, that is, safety set (SS: patients who received the study or comparator treatment and had at least 1 safety assessment). The mean and SD were calculated for continuous data such as patient demographics, health condition, pain intensity (NRS), and EQ-5D, CGIC, and PGIC scores. *T*-tests were used to evaluate the significance of mean differences between the BTDS and TA groups where assumptions of normality were met. Otherwise, the Wilcoxon rank sum tests were used. Paired *t*-tests were used to evaluate the significance of changes in pain score from baseline at each visit for the BTDS and TA groups. Categorical data were summarized with frequencies and percentages and the Chi-square test or Fisher's exact test was used to evaluate the significance of between-group differences. All statistical analyses were performed using SAS™ statistical analysis software (SAS Institute, Cary, NC, USA, version 9.4). Except for noninferiority analysis, statistical significance was evaluated at the 0.05 level.

## 3. Results

**Figure 2:**
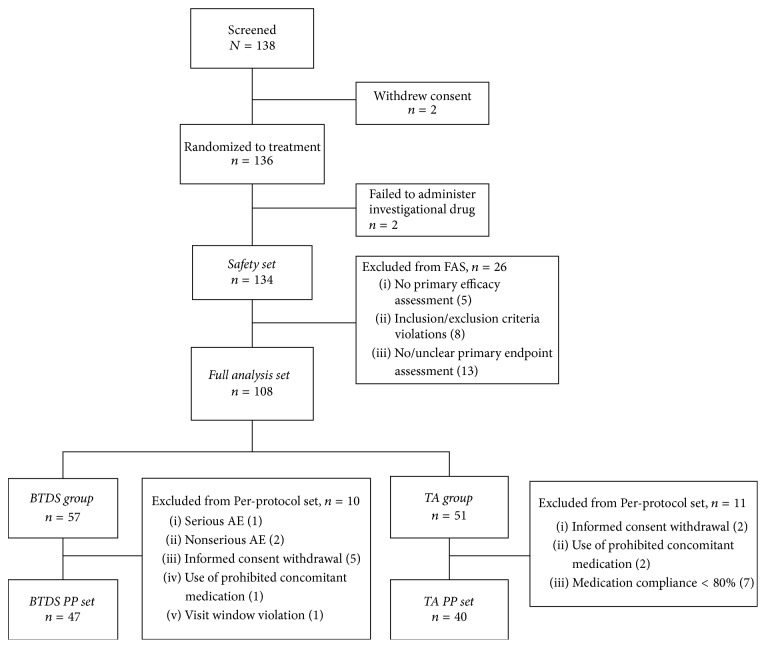
*Flow of patients through the trial*. FAS: full analysis set; BTDS: buprenorphine transdermal system (NORSPAN); TA: tramadol/acetaminophen.

The study was conducted from Oct 2013 to Nov 2014. A total of 138 patients were screened. Of these, 2 withdrew consent and the remaining 136 patients were randomized for treatment. Two subjects failed to receive the investigational treatments. The SS thus consisted of 134 subjects (69 in the BTDS group, 65 in the TA group) who received the study or comparator treatment and had at least 1 safety assessment (Figure 2). Another 26 subjects were excluded from the FAS. The reasons for exclusion and corresponding number of subjects are provided in Figure 2. A total of 87 subjects completed the study (PP set): 47 in the BTDS group and 40 in the TA group.

Subject characteristics and coexisting medical conditions for the SS are shown in Table 2. There were no significant differences between the BTDS and TA groups in terms of patient characteristics (*P* > 0.05 for all; Table 2). The average age for the BTDS group was 64.4 ± 10.8 years and 61.5 ± 9.0 years for the TA group. About 4 in 10 subjects were males. On average, subjects were enrolled approximately 20 days after undergoing lumbar fusion surgery.

### 3.1. Pain Intensity Scores

**Figure 3:**
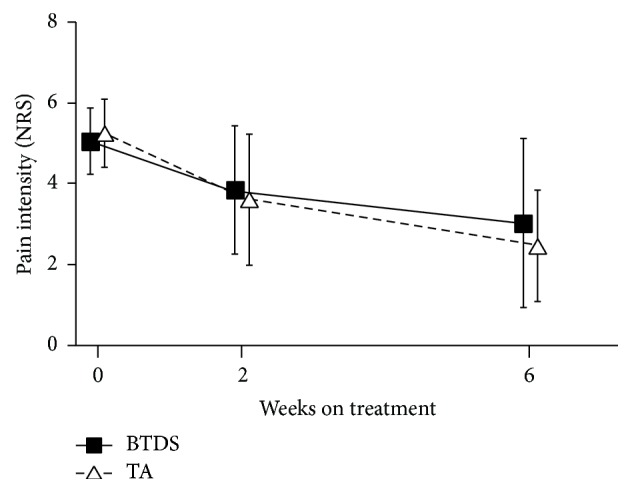
*Mean pain intensity scores from baseline to Week 6*. BTDS: buprenorphine transdermal system (NORSPAN); TA: tramadol/acetaminophen; NRS: numerical rating scale.

On average, patients in both groups reported reduction in pain intensity from “moderate” at baseline (mean NRS: BTDS 5.04 ± 0.82; TA 5.24 ± 0.84) to “mild” at week 6 (mean NRS: BDTS 2.73 ± 1.86; TA 2.49 ± 1.40). At week 2 and at week 6, mean NRS scores were significantly lower than at baseline in both BTDS and TA groups (*P* < 0.0001 for all) (Figure 3). The mean NRS reductions from baseline at week 6 (last observation carried forward, LOCF) were 2.02 ± 2.14 units for the BTDS group and 2.76 ± 1.45 units for the TA group.

At week 6, the between-group difference in mean NRS reduction was 0.74 and the lower limit of the one-sided 97.5% CI of that mean was −1.45 (>−1.5), indicating that BTDS is not inferior to tramadol/acetaminophen for pain reduction. The results for the PP analysis were also in agreement: the mean between-group difference was 0.43 and lower limit of the CI was −1.20 (>−1.5).

### 3.2. Quality of Life and Treatment Satisfaction Scores

Table 3 shows the QoL (EQ-5D-3L) and treatment satisfaction scores (CGIC and PGIC). At week 6, the improvement in mean EQ-5D index scores from baseline was significant for both BTDS and TA groups (*P* < 0.05 for both). The TA group reported a greater improvement in the EQ-5D index score than the BTDS group (*P* = 0.0258). For individual EQ-5D dimension items, significant improvements were seen for “self-care” and “usual activities” for both groups and in “pain/discomfort” for the TA group.

Overall, subjects assessed their condition as somewhat improved after treatment. At week 6, mean CGIC scores in the BTDS and TA groups were 2.28 ± 1.21 and 2.43 ± 1.01, respectively (Table 3). There were no significant between-group differences in terms of treatment satisfaction as assessed by both investigators and subjects at week 6.

### 3.3. Medication Use and Compliance

Table 4 presents medication use and compliance data for the FAS. At Week 2 and Week 6, the BTDS group had a statistically significantly higher compliance rate than the TA group (*P* = 0.078 and <0.0001, resp.).

Overall, 16% of subjects had a dose increase during the study period: 9 subjects from the BTDS group had their dose increased from 5 *μ*g/h to 10 *μ*g/h; and 8 subjects from the TA group had their dose increased from 1 tablet twice daily to 2 tablets twice daily. There were no significant between-group differences in the total dosage of rescue medication used, either in the 2 weeks from baseline to week 2, or in the rest of the study period, from week 2 to week 6.

### 3.4. Adverse Events

The incidence of adverse events was similar in the 2 groups, being 26.1% (18/69 subjects, 31 events) in the BTDS group and 20.0% (13/65 subjects, 22 events) in the TA group (*P* = 0.4037); and similarly for the incidence of serious adverse events: 4.4% (3/69 subjects, 3 events) in the BTDS group while no serious adverse event was reported in the TA group (*P* = 0.2450) (Table 5). There was no significant between-group difference in the incidence rate for adverse drug reactions where a causal relation with the investigational product could not be ruled out (20.3% versus 16.9%, *P* = 0.6171).

In both groups, the most commonly reported AE was gastrointestinal disorders, mainly nausea and constipation (see Table 6, and Supplementary Table  S1, in Supplementary Material available online at https://doi.org/10.1155/2017/2071494, for a detailed list of adverse events). Four subjects receiving BTDS reported general disorders including patch administration site conditions (5 events: administration site pruritus [3 events] and administration site erythema [1 event]), while 3 subjects receiving TA had elevated liver enzymes (AST, ALT; 6 events). Two serious AEs were reported in the BTDS group: “postprocedural infection” at 2.90% (2/69 subjects, 2 events) and “arthralgia” at 1.45% (1/69 subjects, 1 event); no SAEs were reported in the TA group. No serious adverse drug reactions were reported. All adverse events were of grade 1 (mild) or 2 (moderate) in severity. There were no adverse events of grade 3 (severe) or above.

## 4. Discussion

Patients who undergo instrumented spinal fusion and decompression surgery often suffer from severe postoperative pain, which may persist for several weeks [34]. To our knowledge, this study is the first to report on the use of the buprenorphine transdermal patch to manage subacute pain in the 1–3-month period following surgery. In our study, both BTDS and TA reduced the intensity of persistent postoperative pain from “moderate” to “mild” after 6 weeks of treatment. BTDS has been shown elsewhere to be effective for pain control for at least 3 months without significant dose increase [18]. There were no significant differences between the BTDS and TA groups in terms of the incidence of adverse events or adverse drug reactions. For the BTDS group, the most common AEs were gastrointestinal events (including nausea, constipation, and vomiting) and skin reactions at the patch site (including pruritus and erythema). The safety profile for BTDS in this study was consistent with earlier studies on the long-term use of buprenorphine for patients suffering chronic pain [20, 23].

Both clinicians and subjects assessed their condition as somewhat improved since the start of treatment; the extent of improvement was similar in the BTDS and TA groups. Importantly, subjects reported significant improvements in the “self-care” and “usual activities” EQ-5D dimensions, suggesting that they felt better able to manage their usual activities on their own after 6 weeks of treatment.

The management of persistent postoperative pain is associated with a number of challenges. Achieving adequate pain control is important to limit the negative effects of persistent postoperative pain, but inappropriate use of strong opioids to manage pain is associated with a risk of tolerance and abuse [35] and, in the elderly, delirium and cognitive decline [36]. The results of a systematic review on long-term opioid management of chronic noncancer pain suggest that many patients discontinue treatment due to adverse events or insufficient pain relief [37]. Certain groups of patients may be particularly affected by the adverse side-effects of opioids, and may require proactive management [38]. For example, opioid doses for older patients may have to be reduced to half of the standard adult dose in order to limit adverse events while maintaining an adequate level of analgesia [8]. Buprenorphine, on the other hand, does not require dose adjustments for those with impaired renal function or for the elderly [39]. In this study, subjects who received BTDS reported mainly gastrointestinal-related adverse events such as nausea and constipation. These side-effects are typical of opioid analgesics, though rates of constipation associated with buprenorphine are reported to be lower than for other opioids [22]. Other commonly reported events for patients receiving BTDS included erythema (*n* = 1; 1.5%) or pruritus (*n* = 3; 4.4%) at the patch application site (reported in the “General disorders and administration site conditions” category, Table 6; Supplementary Table  S1). Application site reactions are commonly reported with other transdermal opioid formulations, for example, fentanyl patches. Respiratory depression is a potentially life-threatening adverse event associated with use of opioids. However, the risk of respiratory depression is lower with buprenorphine due to its “respiratory ceiling” effects [22]. The dose against time effect plot for intravenous buprenorphine exhibits a “ceiling” at doses ≥ 0.2 mg. This “ceiling effect,” coupled with the lack of apnea at high doses, makes buprenorphine a much safer drug to use. At steady state, elderly (≥75 years) and younger (50–60 years) patients who were systemically exposed to buprenorphine showed similar efficacy and safety profiles despite the expected age-related pharmacodynamic and pharmacokinetic differences in physiology [39, 40].

Some limitations of our study should be considered when interpreting the results. Increase in pain medication dose was permitted during the study, similar to normal clinical practice, in which doses would be adjusted for individual patients to achieve an acceptable level of pain control. However, the equivalent efficacy of initial doses for the study and control treatments could not be determined at the outset. We therefore selected the lowest available doses (i.e., 5 *μ*g/h BTDS and 1 tablet of tramadol 37.5 mg/acetaminophen 325 mg twice daily). We also note that the possibility of dose increases could make it more difficult to interpret comparisons between the groups. Nevertheless, the observed proportion of subjects with a dose increase was very similar in the 2 groups (BTDS: 17.5%; TA: 17.0%). Moreover, the actual dose increases in both groups were modest, from 5 *μ*g/h to 10 *μ*g/h for BTDS and from 1 tablet twice daily to 2 tablets twice daily for TA. Only 8 subjects in the BTDS group and 7 in the TA group had their prescribed pain medication up-titrated even though there were many subjects with moderate pain in the second week (mean NRS scores were 3.9 ± 1.6 and 3.5 ± 1.6, resp.). This implies that active up-titration of the medication dose may not have taken place for some subjects. For BTDS, it is possible that the longer dosing interval and slower titration (no more than once every 7 days) could have led to fewer subjects having their medication up-titrated. These characteristics should be taken into account when designing future studies involving BTDS therapy. Despite the small number of up-titration cases, the mean NRS scores recorded at week 6 were below 3 for both treatments (BTDS 2.7 ± 1.9; TA 2.5 ± 1.5), which is considered a meaningful level of pain control [41].

A limitation of a nonblinded design is the potential for bias in subjective assessments even when an active comparator is used. As the aim was to evaluate the strengths and weaknesses of the treatments under normal clinical use and according to product prescribing information, an open-label active-control study design was selected. The different characteristics of the 2 treatments (e.g., administration route, dosing interval, and speed of titration) may have implications for patient preference and compliance and would need to be taken into consideration by physicians when individualizing treatment for patients. As transdermal buprenorphine is not recommended for patients with rapidly changing analgesic requirements, subjects with severe or unstable pain would have been underrepresented in this study. Thus, our conclusions may not be generalizable to patients with more severe pain.

While both groups of subjects showed statistically significant improvement in most of the parameters evaluated, the compliance rate was higher among subjects using the buprenorphine patch than among those receiving tramadol/acetaminophen tablets. Similar observations were made by Lovborg et al. in their review of transdermal opioid patches [42]. All subjects who received the BTDS patch met the minimum compliance rate of 80% at the end of 6 weeks; 7 subjects in the TA group were excluded from the PP set for poor compliance, leading to a lower than expected number of subjects in the TA group completing the study without protocol deviations. This result suggests that the use of a once-weekly transdermal patch may be more convenient for prolonged use compared to twice-daily tablets, especially in older patients who have coexisting medical conditions and are concurrently on multiple oral medications. For these patients, the buprenorphine patch system does not add to the number of pills to be taken daily and can help to improve compliance when the medication has to be taken for several weeks.

## 5. Conclusions

The buprenorphine transdermal system was comparable to oral tramadol/acetaminophen in reducing pain intensity after 6 weeks of treatment in patients with persistent moderate postoperative pain at 1–3 months after spinal surgery. Patients treated with BTDS also had better compliance to the prescribed treatment.

## Supplementary Material

Supplementary Table S1. Details of adverse events listed by System Organ Class for the Safety Set.

## Figures and Tables

**Table 1:** Computation of EQ-5D index score.

Item^a^	Level 1	Level 2	Level 3
Mobility	0	0.069	0.314
Self-care	0	0.104	0.214
Usual activities	0	0.036	0.094
Pain/discomfort	0	0.123	0.386
Anxiety/depression	0	0.071	0.236

^a^Mobility: Level 1: I have no problems in walking around; Level 2: I have some problems in walking around; Level 3: I am confined to bed. Self-care: Level 1: I have no problems with self-care; Level 2: I have some problems taking a bath/shower, or dressing myself; Level 3: I am unable to take a bath/shower, or dress myself. Usual activities: Level 1: I have no problems with performing my usual activities; Level 2: I have some problems with performing my usual activities; Level 3: I am unable to perform my usual activities. Pain/discomfort: Level 1: I have no pain or discomfort; Level 2: I have moderate pain or discomfort; Level 3: I have extreme pain or discomfort. Anxiety/depression: Level 1: I am not anxious or depressed; Level 2: I am moderately anxious or depressed; Level 3: I am extremely anxious or depressed. The EQ-5D index (total score) was computed as follows: 1 − 0.081 − (score according to item and level) − 0.269 (if there was at least one Level 3 item).

**Table 2:** Patient characteristics and medical conditions.

Safety set	Buprenorphine transdermal system (*n* = 69)	Oral tramadol/acetaminophen (*n* = 65)	*P* value^a^
Sex, male, *n* (%)	25 (36.2)	29 (44.6)	0.3228
Age, mean ± SD, years	64.4 ± 10.8	61.5 ± 9.0	0.0969
Age distribution, *n* (%)			
≥70 years, *n* (%)	24 (34.8)	11 (16.9)	0.0637
60–69 years, *n* (%)	27 (39.1)	30 (46.1)	
50–59 years, *n* (%)	12 (17.4)	20 (30.8)	
<50 years, *n* (%)	6 (8.7)	4 (6.1)	
Weight (baseline), mean ± SD, kg	62.2 ± 9.4	62.0 ± 10.2	0.9176
Period after lumbar fusion surgery, mean ± SD, days	20.9 ± 11.8	19.2 ± 9.5	0.3532
10 days to <1 month, *n* (%)	61 (88.4)	57 (87.7)	0.8987
≥1 month, *n* (%)	8 (11.6)	8 (12.3)	
History of medical or allergy condition over past 2 years, *n* (%)	55 (79.7)	57 (87.7)	0.2125
Used prior medication within 4 weeks of baseline visit, *n* (%)	68 (98.6)	64 (98.5)	1.0000
Had ≥1 dose of analgesics or drugs with analgesic effect during the study, other than study or rescue medication	7 (10.1)	7 (10.8)	0.9060

^a^Chi-square or Fisher's exact test.

**Table 3:** Quality of life and treatment satisfaction scores.

Full analysis set	Buprenorphine transdermal system (*n* = 47)	Oral tramadol/acetaminophen (*n* = 40)	*P* value (*T*-test)
EQ-5D index^a^, mean ± SD			
Baseline (week 0)	0.57 ± 0.13	0.46 ± 0.27	—
Week 6 (LOCF)	0.68 ± 0.18	0.65 ± 0.14	—
Improvement (week 0 to week 6)	0.10 ± 0.19	0.19 ± 0.21	**0.0258**
*P* value (paired *t* test)	**0.0004**	**<0.0001**	—
EQ-5D-3L dimension scores			
(1) Mobility			
Baseline (week 0)	0.86 ± 0.08	0.85 ± 0.11	0.7111
Week 6 (LOCF)	0.89 ± 0.03	0.88 ± 0.03	0.6256
Improvement (week 0 to week 6)	0.03 ± 0.08	0.03 ± 0.11	0.8326
*P* value (paired *t* test)	**0.0080**	**0.0281**	
(2) Self-care			
Baseline (Week 0)	0.83 ± 0.07	0.78 ± 0.14	0.0161
Week 6 (LOCF)	0.87 ± 0.05	0.84 ± 0.07	0.0108
Improvement (week 0 to week 6)	0.04 ± 0.08	0.06 ± 0.13	0.3247
*P* value (paired *t* test)	**0.0008**	**0.0021**	
(3) Usual activities			
Baseline (week 0)	0.88 ± 0.01	0.82 ± 0.14	0.0007
Week 6	0.89 ± 0.02	0.89 ± 0.05	0.6431
Improvement (Week 0 to Week 6)	0.01 ± 0.01	0.07 ± 0.13	**0.0006**
*P* value (paired *t* test)	**0.0019**	**0.0002**	
(4) Pain/discomfort			
Baseline (week 0)	0.80 ± 0.02	0.78 ± 0.10	0.1245
Week 6 (LOCF)	0.81 ± 0.09	0.82 ± 0.05	0.6831
Improvement (week 0 to week 6)	0.01 ± 0.09	0.04 ± 0.12	0.1731
*P* value (paired *t* test)	0.2940	**0.0158**	
(5) Anxiety/depression			
Baseline (Week 0)	0.88 ± 0.04	0.88 ± 0.08	0.8932
Week 6 (LOCF)	0.89 ± 0.07	0.90 ± 0.03	0.5537
Improvement (week 0 to week 6)	0.01 ± 0.08	0.02 ± 0.07	0.6613
*P* value (paired *t* test)	0.1865	**0.0479**	
Treatment satisfaction scores at week 6, mean ± SD			
CGIC^b^	2.46 ± 1.28	2.37 ± 0.98	0.7067
PGIC^b^	2.53 ± 1.28	2.39 ± 0.92	0.5304

^a^Quality of life (EQ-5D-3L, Korean version): consists of 5 items, that is, mobility, self-care, normal activities, pain/discomfort, and anxiety/depression. EQ-5D-3L scores were computed according to Table 1; ^b^CGIC, PGIC: Clinical Global Impression of Change, Patient Global Impression of Change. 1 = very much improved since the initiation of treatment; 2 = much improved; 3 = minimally improved; 4 = no change from baseline (the initiation of treatment); 5 = minimally worse; 6 = much worse; 7 = very much worse since the initiation of treatment. *P* values < 0.05 are marked in bold text.

**Table 4:** Medication compliance rate, dose change, and use of rescue medication.

Full analysis set	Buprenorphine transdermal system (*N* = 57)	Oral tramadol/acetaminophen (*N* = 51)	*P* value
Medication compliance rate^*∗*^, %, mean ± SD			
At Week 2	97.9 ± 10.1	92.5 ± 21.1	**0.0078** ^†^
At Week 6	97.8 ± 14.1	91.0 ± 18.9	**<0.0001** ^†^
Dose change of study medication			
Patients requiring dose change during study, *n* (%)	9 (15.8)	8 (15.7)	—
Week 6 dose of patients who had dose change	10 *μ*g/h	2 tablets	—
Dose change, mean ± SD	0.79 ± 1.84 *μ*g	0.16 ± 0.37 tablets	—
Total dose of rescue medication, mg, mean ± SD			
Baseline to week 2 (2-week duration)	1,439.2 ± 1,741.3	1,412.2 ± 2,009.7	0.9429^‡^
Week 2 to week 6 (4-week duration)	1,534.7 ± 1,820.0	1,869.4 ± 2,530.0	0.4542^‡^

^*∗*^Medication compliance rate = total number of doses actually administered (actual number of medication days)/total number of doses prescribed (number of medication days prescribed); ^†^Wilcoxon rank sum test; ^‡^*T*-test.

**Table 5:** Adverse events.

Safety set	Group	Subjects, *n*	Number of events, *n*	Incidence, *n* (%)	95% CI (lower, upper)	*P* value
Adverse events (AE)	BTDS	69	31	18 (26.1)	(15.7, 36.5)	0.4037^a^
	TA	65	22	13 (20.0)	(10.3, 29.7)	
	Total	134	53	31 (23.1)	(16.0, 30.3)	

Serious adverse event (SAE)	BTDS	69	3	3 (4.4)	(0.0, 9.2)	0.2450^b^
	TA	65	0	0 (0)	—	
	Total	134	3	3 (2.2)	(0.0, 4,7)	

Adverse drug reaction (ADR)	BTDS	69	19	14 (20.3)	(10.8, 29.8)	0.6171^a^
	TA	65	13	11 (16.9)	(7.8, 26.0)	
	Total	134	32	25 (18.7)	(12.1, 25.3)	

Withdrawals due to AE	BTDS	69	5	4 (5.8)	(0.3, 11.3)	0.1201^b^
	TA	65	0	0 (0)	—	
	Total	134	5	4 (3.0)	(0.1, 5.9)	

^a^Chi-square test; ^b^Exact test; BTDS: buprenorphine transdermal system; TA: tramadol/acetaminophen.

**Table 6:** Adverse events by system organ class.

Safety set	Buprenorphine transdermal system		Oral tramadol/acetaminophen		Total	
	Subjects (*N* = 69)	Events	Subjects (*N* = 65)	Events	Subjects (*N* = 134)	Events
Gastrointestinal disorders	12 (17.4)	14	9 (13.9)	9	21 (15.7)	23
General disorders and administration site conditions	4 (5.8)	5	1 (1.5)	1	5 (3.7)	6
Nervous system disorders	4 (5.8)	4	—	—	4 (3.0)	4
Musculoskeletal and connective tissue disorders	3 (4.4)	4	1 (1.5)	1	4 (3.0)	5
Investigations	1 (1.5)	1	3 (4.6)	6	4 (3.0)	7
Skin and subcutaneous tissue disorders	—	—	3 (4.6)	3	3 (2.2)	3
Infections and infestations	2 (2.9)	2	1 (1.5)	1	3 (2.2)	3
Cardiac disorders	—	—	1 (1.5)	1	1 (0.8)	1
Injury, poisoning and procedural complications	1 (1.5)	1	—	—	1 (0.8)	1

*Total* ^*a*^	*18 (26.1)*	*27*	*13 (20.0)*	*22*	*31 (23.1)*	*53*

^a^All adverse events were of severity grades 1 (mild) or 2 (moderate). There were no adverse events of severity greater than grade 3 (severe).
