# Supplementary material for: Efficacy and Safety of Transdermal Buprenorphine versus Oral Tramadol/Acetaminophen in Patients with Persistent Postoperative Pain after Spinal Surgery
Source: Pain Res Manag. 2017 Sep 13;2017:2071494. doi: 10.1155/2017/2071494 (PMC5615987; doi:10.1155/2017/2071494)
Supplement: Supplementary file 1 — Supplementary Table S1. Details of adverse events listed by System Organ Class for the Safety Set. [file 2071494.f1.docx]

Supplementary Table S1: All adverse events^a^

|  | Buprenorphine  transdermal system | | | Tramadol/ acetaminophen | | | Total | | |
| --- | --- | --- | --- | --- | --- | --- | --- | --- | --- |
|  | **Incidence** | | **Events** | **Incidence** | | **Events** | **Incidence** | | **Events** |
| Safety Set | **N=69** | **%** | **n** | **N=65** | **%** | **n** | **N=134** | **%** | **n** |
| Gastrointestinal disorders | 12 | 17.39 | 14 | 9 | 13.85 | 9 | 21 | 15.67 | 23 |
| Constipation | 4 | 5.80 | 4 | 4 | 6.15 | 4 | 8 | 5.97 | 8 |
| Nausea | 5 | 7.25 | 6 | 3 | 4.62 | 3 | 8 | 5.97 | 9 |
| Dyspepsia | 0 | 0.00 | 0 | 2 | 3.08 | 2 | 2 | 1.49 | 2 |
| Vomiting | 2 | 2.90 | 2 | 0 | 0.00 | 0 | 2 | 1.49 | 2 |
| Diarrhoea | 1 | 1.45 | 1 | 0 | 0.00 | 0 | 1 | 0.75 | 1 |
| Gastric disorder | 1 | 1.45 | 1 | 0 | 0.00 | 0 | 1 | 0.75 | 1 |
| General disorders and administration site conditions | 4 | 5.80 | 5 | 1 | 1.54 | 1 | 5 | 3.73 | 6 |
| Application site pruritus | 3 | 4.35 | 3 | 0 | 0.00 | 0 | 3 | 2.24 | 3 |
| Application site erythema | 1 | 1.45 | 1 | 0 | 0.00 | 0 | 1 | 0.75 | 1 |
| Chills | 1 | 1.45 | 1 | 0 | 0.00 | 0 | 1 | 0.75 | 1 |
| Oedema | 0 | 0.00 | 0 | 1 | 1.54 | 1 | 1 | 0.75 | 1 |
| Investigations | 1 | 1.45 | 1 | 3 | 4.62 | 6 | 4 | 2.99 | 7 |
| Alanine aminotransferase increased | 0 | 0.00 | 0 | 3 | 4.62 | 3 | 3 | 2.24 | 3 |
| Aspartate aminotransferase increased | 0 | 0.00 | 0 | 3 | 4.62 | 3 | 3 | 2.24 | 3 |
| Haemoglobin decreased | 1 | 1.45 | 1 | 0 | 0.00 | 0 | 1 | 0.75 | 1 |
| Musculoskeletal and connective tissue disorders | 3 | 4.35 | 4 | 1 | 1.54 | 1 | 4 | 2.99 | 5 |
| Arthralgia | 1 | 1.45 | 1 | 1 | 1.54 | 1 | 2 | 1.49 | 2 |
| Costochondritis | 1 | 1.45 | 1 | 0 | 0.00 | 0 | 1 | 0.75 | 1 |
| Myalgia | 1 | 1.45 | 2 | 0 | 0.00 | 0 | 1 | 0.75 | 2 |
| Nervous system disorders | 4 | 5.80 | 4 | 0 | 0.00 | 0 | 4 | 2.99 | 4 |
| Dizziness | 2 | 2.90 | 2 | 0 | 0.00 | 0 | 2 | 1.49 | 2 |
| Headache | 1 | 1.45 | 1 | 0 | 0.00 | 0 | 1 | 0.75 | 1 |
| Paraesthesia | 1 | 1.45 | 1 | 0 | 0.00 | 0 | 1 | 0.75 | 1 |
| Infections and infestations | 2 | 2.90 | 2 | 1 | 1.54 | 1 | 3 | 2.24 | 3 |
| Post procedural infection | 2 | 2.90 | 2 | 0 | 0.00 | 0 | 2 | 1.49 | 2 |
| Herpes zoster | 0 | 0.00 | 0 | 1 | 1.54 | 1 | 1 | 0.75 | 1 |
| Skin and subcutaneous tissue disorders | 0 | 0.00 | 0 | 3 | 4.62 | 3 | 3 | 2.24 | 3 |
| Pruritus | 0 | 0.00 | 0 | 1 | 1.54 | 1 | 1 | 0.75 | 1 |
| Pruritus generalised | 0 | 0.00 | 0 | 1 | 1.54 | 1 | 1 | 0.75 | 1 |
| Urticaria | 0 | 0.00 | 0 | 1 | 1.54 | 1 | 1 | 0.75 | 1 |
| Cardiac disorders | 0 | 0.00 | 0 | 1 | 1.54 | 1 | 1 | 0.75 | 1 |
| Palpitations | 0 | 0.00 | 0 | 1 | 1.54 | 1 | 1 | 0.75 | 1 |
| Injury, poisoning and procedural complications | 1 | 1.45 | 1 | 0 | 0.00 | 0 | 1 | 0.75 | 1 |
| Post procedural swelling | 1 | 1.45 | 1 | 0 | 0.00 | 0 | 1 | 0.75 | 1 |
| Total | **18** | **26.09** | **31** | **13** | **20.00** | **22** | **31** | **23.13** | **53** |

^a^ All events were of grades 1 (mild) or 2 (moderate) in severity. There were no adverse events of severity of grade 3 (severe) or greater.

^b^ Coding Dictionary: MedDRA
